# 2q31 microdeletion syndrome with the velocardiofacial phenotype and review of the literature: a case report

**DOI:** 10.1186/s12887-024-04843-7

**Published:** 2024-10-09

**Authors:** Estephania Candelo, Sebastian Giraldo-Ocampo, Julian Nevado, Pablo Lapunzina, Harry Pachajoa

**Affiliations:** 1 Fundacion Valle del Lili, Cali, Colombia; 2 Congenital and rare disease center (CIACER), Universidad Icesi, Cali, Colombia; 3 Hospital Universitario La Paz, Madrid, Spain; 4 Genetics Division, Fundación Valle del Lili, Carrera 98 # 18-49, Cali, Colombia

**Keywords:** Facial dysmorphism, 2q31-q32 microdeletion, Array CGH, Velocardiofacial syndrome

## Abstract

**Background:**

The 2q31 deletion results in a distinct phenotype characterized by varying degrees of developmental delay, short stature, facial dysmorphism, and variable limb defects. Dysmorphic features include microcephaly, downslanting palpebral fissures, a long and flat philtrum, micrognathia, and dysplastic, low-set ears. To date, comparative genomic hybridization has identified this deletion in 38 patients. Consequently, additional patients with comprehensive clinical data are required to fully understand the spectrum of clinical manifestation associated with a deletion in the 2q31 cytoband.

**Case presentation:**

We present the case of an 8-year-old female patient with clinical features of velocardiofacial syndrome, which include facial dysmorphism, congenital heart disease (persistent truncus arteriosus and ostium secundum-type atrial septal defect), and a seizure syndrome. Array comparative genomic hybridization revealed a non-continous deletion spanning cytobands 2q31.1-to 2q31.3, confirming a diagnosis of 2q31 microdeletion syndrome. The patient has undergone supportive therapies for swallowing and speech. Additionally, we provide a review of the literature on previous cases to give context.

**Conclusion:**

In this report, we present the first documented case of a complex, discontinuous deletion spanning in the 2q31-2q32 regions. This case contributes to our understanding of the phenotypic and mutational spectrum observed in individuals with deletions in these cytobands. It underscores the significance of employing high-resolution techniques and comprenhensive analysis in diagnosing patients with complex phenotypes. Such approaches are crucial for differentiating this condition from more common microdeletion syndromes, such as the 22q11 deletion syndrome.

## Introduction

2q31 microdeletion syndrome, not described in the Online Mendelian Inheritance in Man (OMIM) database except for the 2q31.2 deletion syndrome (OMIM #612,345), presents with a distinctive clinical phenotype. This syndrome phenotype is characterized by varying degrees of development delay, short stature, facial dysmorphism, and variable limb defects [1]. The dysmorphic features typically include microcephaly, downslanting palpebral fissures, a long and flat philtrum, micrognathia, and dysplastic low set-ears. Extremity abnormalities include ectrodactyly, brachydactyly, syndactyly, and camptodactyly [2, 3]. 

To date, 38 cases have been analyzed using comparative genomic hybridization (CGH) microarray [2, 4]. These deletions typically span the cytobands 2q31 and 2q32, with some extending into the 2q33 region. Establishing a genotype-phenotype correlation has proven challenging, as patients with similar deletions often exhibit different phenotypes [2, 4]. . Prior studies have identified candidate genes responsible for certain characteristic features of this syndrome, such as the *ZNF385B* and the *HOXD* gene cluster [5–7]. However, to better characterize the full clinical spectrum of 2q31 microdeletion syndrome, there is a need for more cases and comprehensive clinical data. This data can help explore the potential genotype-phenotype correlations, understand the impact of deletion size on disease severity, and identify new critical genes or regions associated with the observed clinical manifestations. In this context, we present the first case of a Colombian patient exhibiting the phenotype of velocardiofacial syndrome, with a non-continuous heterozygous deletion on chromosome 2 that includes the 2q31 and 2q32 cytobands. This case not only enhances our understanding of this exceptionally rare syndrome but also underscores the importance of high-resolution techniques, such as CGH microarray, in accurate diagnosis of patients with complex phenotypes.

## Case presentation

The proband is an 8-year-old female patient with an unknown family history who experienced intrauterine growth restriction. She was born prematurely at 28 weeks, with a birth weight of 1700 g (99.6th percentile). At six months after birth, she presented with hypothyroidism and received appropriate pharmaceutical treatment. At seven months of age, she developed status epilepticus, requiring eight days of intensive care; she was also diagnosed with congenital heart disease, characterized by persistent truncus arteriosus (type II) and interatrial communication (ostium secundum type). Surgical correction of the cardiac abnormalities was performed at nine months of age.

**Fig. 1:**
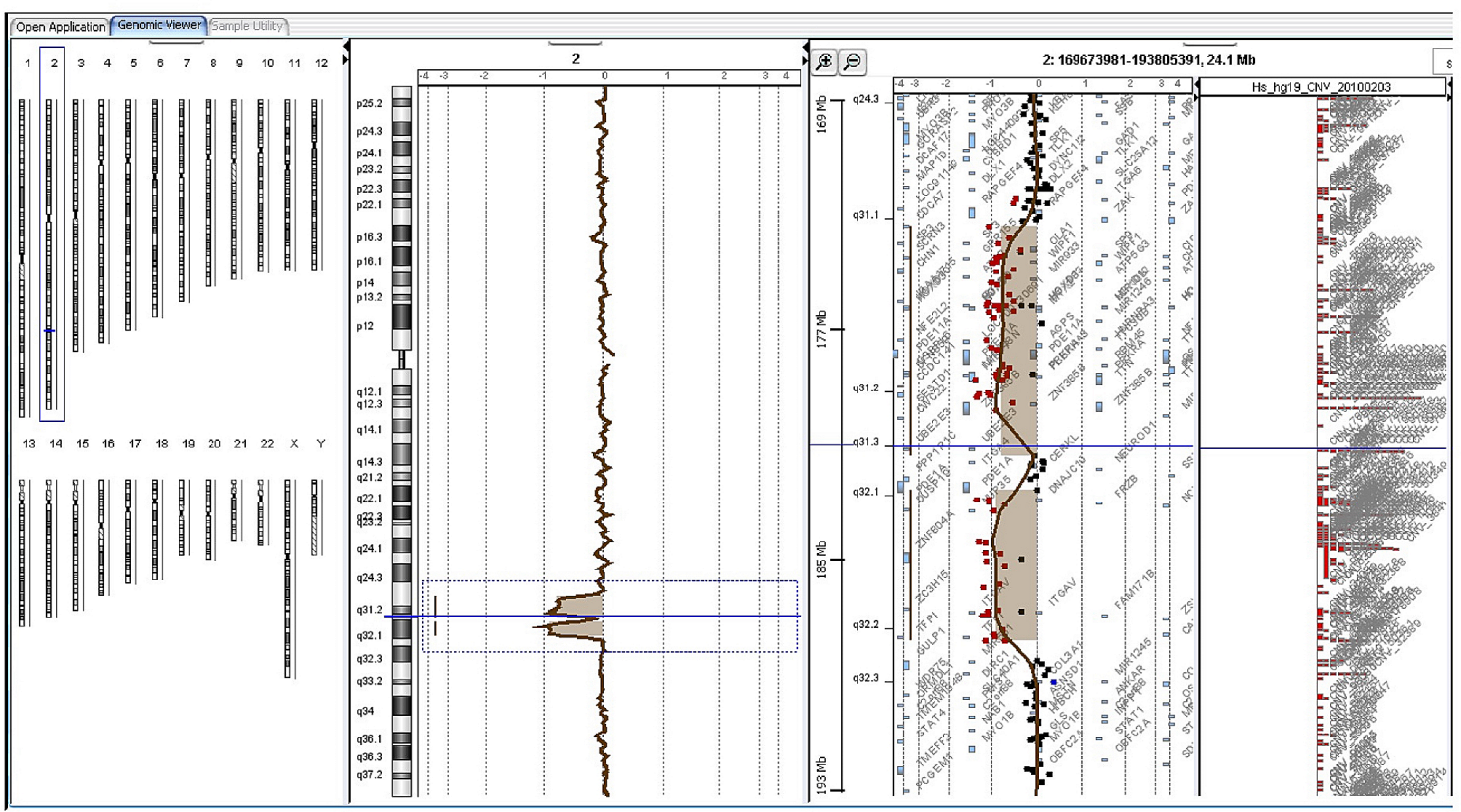
A representation of the aCGH results, which include the first deleted region of approximately 7.89 Mb of DNA localized on 2q31.1-q31.2, and the second deleted region of approximately 5.23 Mb on the cytoband 2q32.1

At 4 years old, she underwent her initial evaluation by clinical genetics, presenting with a height, weight, and head circumference of 87 cm (-3.79 SD), 8.65 kg (− 4.5 SD), and 43.5 cm (-4.1 SD), respectively. The physical examination revealed profound developmental delay, absence of speech, thick and coarse hair, a triangular forehead hair pattern, thick eyebrows, synophrys, microcephaly, facial asymmetry, a thin nasal bridge, a bifid nasal tip, dysmorphic ears, strabismus, low set and posteriorly rotated ears with prominent antihelix, micrognathia, and a right equinovarus deformity that was correctable both actively and passively. Additional findings included bilateral dimples on the elbows and knees, sacral dimples, a unique right palmar crease, bilateral camptodactyly, brachydactyly of the third and fourth finger on the right hand and the fourth finger of the left hand, hypotonia, pectus excavatum, well-defined musculature, aggression and disorganized movements (Fig. 1).

Complementary studies indicated acetabular dysplasia, and lumbosacral ultrasound revealed cutaneous anomalies without evidence of spinal dysraphism. Post-Rastelli procedure ecography demonstrated normal biventricular function, a bilateral pulmonary lesion characterized by mild pulmonary stenosis, and mild aortic regurgitation without hemodynamic consequences. The patient has received only supportive therapy for oropharyngeal dysphagia and speech disturbances. Currently, the patient’s speech is characterized by babbling and the utterance of syllabes, associated with a significant masticatory deficit and residual material in the pharyngeal recesses, specifically the valleculae and pyriform sinuses. These deficits, which as identified were in a videofluoroscopic swallowing study, have led to multiple pulmonary infections from bronchoaspiration. Additionally, the patient exhibits severe global developmental delay and is experiencing acute, mild-to-moderate malnutrition.

Furthermore, she has been diagnosed with obstructive sleep apnea and hypopnea syndromes, which are being managed with continuous positive airway pressure (CPAP) therapy. Magnetic resonance imaging (MRI) revealed dysgenesis of the corpus callosum, encephalomalacia in the right frontal lobe, retraction of the frontal pole of the lateral ventricle, and periventricular hyperintensities. Karyotyping showed a normal female karyotype (46, XX). Fluorescence in situ hybridization (FISH) analyses for subtelomeric rearrangements and deletions in 22q11, conducted to rule out a microdeletion syndrome, yielded negative results. Our study utilized the high-resolution oligonucleotide-based 2 × 400 array-CGH technique on the Agilent platform. Following the experimental procedure, we generated a Cyto Report file and used Agilent Cytogenomics software version 3.0.6.6 for data analysis. During this analysis, we consulted various genetic databases, including the Database of Genomic Variants (DGVs), the UCSC Genome browser for the human February 2009 release (GRCh 37/hg19 Assembly), and the Database of Chromosomal Imbalance and Phenotype in Humans Using Ensemble Resources (DECIPHER).

The SurePrint G3 Human CNV 2 × 400 K Oligo Microarray kit, which provides comprehensive coverage of known CNVs, was utilized in this study. We followed a modified version of Agilent’s procedures. In summary, genomic DNA was extracted, amplified, and purified using the QIAprep Miniprep Kit from QIAGEN, according to the instructions provided by the manufacturer. The concentration of the DNA was determined using a Nano-Drop Spectrophotometer. Cy3-dUTP was used to label the test DNA, while Cy5-dUTP was used to label sex-matched human genomic DNA as the reference. The labeled test and reference DNA samples were then combined, purified, and applied to the microarray chips. The hybridization process was perform according to the manufacturer’s instructions. Microarray images were captured using the Agilent G2505C scanner (HD) and analyzed with the Feature Extraction Software v3.0.5.1 from Agilent Technologies, based in Santa Clara, CA, USA. The analysis utilized specific parameters, which included settings the genome version to hg18, applying aberration filters with a minimum of three probes, requiring a minimum average absolute log ratio of 0.25 for DNA copy number changes, allowing for a maximum of 100,000 aberrations, and a penetrance threshold set at 0%.

Our array comparative genomic hybridization (aCGH) microarray analysis identified two deletions in chromosome 2. The first deletion, located in the cytobands 2q31.1-q31.3, spans an approximately 7.89 Mb region from 174,230,303 to 182,123,207 and affects roughly 48 genes. The second deletion, in the 2q32.1 cytoband, encompasses an area of about 5.23 Mb from 183,291,363 to 188,498,365 and includes 13 deleted (see Fig. 1).

## Discussion

The 2q31.1 microdeletion syndrome, characterized by a wide spectrum of signs and symptoms, is well-documented in the literature. This syndrome is linked to haploinsufficiency of specific genes, which significantly contributes to the variety of clinical presentations observed. An in-depth review of cases reported from 2006 to 2022 identified a total of 39 patients, including the proband in this study, with deletions in the genomic region extending from 2q31.1 to 2q33. The deletions were confirmed using high-resolution aCGH, a technique known for its enhanced resolution and accuracy in detecting genomic anomalies [1, 2, 4–14]. 

In the cases reported, 64% of the patients (16 out of 25 cases with reported gender) were female. The median age at diagnosis was 5.25 years, with an interquartile range (IQR) of 2 to 13.8 years. Approximately half of the patients (51%, 19 out of 37 cases) were identified as having cognitive impairment of varying degrees, with 35% of these cases classified as severe. Seizures were noted in 35.3% of the patients (12 out of 34), and 42.3% exhibited absent or abnormal speech (15 out of 35). Developmental or growth delays were prevalent in 73% of cases (27 out of 30), and an abnormal head shape, most commonly microcephaly, was observed in 64% (23 out of 36). Heart abnormalities were present in 41.2% (7 out of 17), and eye abnormalities in 66.7% (24 out of 36). The eye conditions varied included astigmatism, deep-set eyes, downslanting palpebral fissures, strabismus, coloboma, and lacrimal duct abnormalities. Furthermore, all patients exhibited at least one dysmorphic facial feature, which included a high or prominent forehead, ear anomalies (most often low-set ears), micrognathia, various nasal and dental anomalies (such as crowded, broad, and small teeth), and different palate abnormalities, including cleft palate, ogival palate, high, or narrow palates.

In summary, patients with the 2q31.1 microdeletion syndrome, as documented in the literature, exhibit a broad spectrum of clinical manifestation. These are closely linked to gene haploinsufficiency. The use of high-resolution aCGH has been instrumental in identifying and characterizing deletions within the 2q31.1-2q33 region. This advanced technique enables a detailed analysis of the clinical presentations and the underlying genetic factors contributing to the syndrome.

The median size of deletions in reported cases of 2q31.1 microdeletion syndrome was found to be 5.25 Mb, with an interquartile range (IQR) extending from 2.6 Mb to 11.6 Mb (Fig. 1). Commonly affected genes within these deletions include the HOXD and ZNF clusters, as well as the GLS, FRZB, and SATB2 genes (Table 1). In this study, we report a case involving an infant with a deletion spanning the 2q31-2q32 cytobands, which is larger than the median size at 13.12 Mb. The patient exhibited a range of symptoms, including developmental delay, behavioral and speech issues, seizures, growth retardation, and mild facial dysmorphism (Table 1).

**Table 1:** Clinical characteristics of patients reported in the literature carrying heterozygous microdeletions comprising at least the 2q31.1 region. + and - indicates presence or absence of symptoms, respectively

	Our case	Svensson et al., 2007^7^	Mencarelli et al., 2007^1^	Monfort et al., 2008^11^	Tsai et al., 2009^6^	Rifai et al., 2009^12^	Prontera et al., 2009^13^	Mitter et al., 2010^4^ (8 subjects)	Cocchella et al., 2010^14^	Theisen et al., 2011^2^ (14 subjects)	Ferreira et al., 2012^8^ (Subject #1)	Wang et al., 2014^9^	Puvabanditsin et al., 2015^10^	Okamoto et al., 2017^5^	Dimitrov et al., 2011^3^ (5 subjects)	Total reported	Observation
**Deleted region**	2q31-2q32	2q31.1	2q31.2-q32.3	2q31.2	2q31.1-31.2	2q31.2-q33.2	2q31.2 - q32.3	*see Fig. 2*	2q31.2-q32.3	*see Fig. 2*	2q31.2-q32.3	2q31.1	2q31.1-q33.1	2q31.1-q31.3	2q31.1	39	2q31.1 to 2q33.2
**Deletion size (Mb)**	13.1	2	13	3.3	3.4	26.3	13.7	Median: 2,29 (IQR:1,47 − 6)	4.4	Median: 6,69 (IQR: 2,77 − 10,2)	16.8	3,6	23	6	ND	34	Median:5,25 (IQR:2,6–11,6)
**Sex**	Female	Female	Male	Male	Female	Female	Male	Male: 2; female: 6	Female	ND	Female	Male	Male	Male	Male: 1; Female: 4	25	9 Male/16 Female
**Age (years)**	8	16	13	14	4	16	36	Median: 1,9 (IQR: 1,35 − 5,75)	25	Median: 6 (IQR: 3,25 − 12,5)	8	4	Neonate	2	Median: 4,5 (IQR: 2–6)	39	Median: 6 (IQR:2–13,8)
**Weight at birth (grams)**	1700	3374	1560	ND	2400	2340	2150	Median: 2525 (IQR:1945–2717)	3600	ND	2470	ND	1575	2600	S1: 3500; S2: 1900; S3: 3000; ND(S4,S5)	22	Median: 2430 (IQR:1900–2920)
**Mental Retardation**	Severe	Mild	Severe	Mild	Mild	Severe	Severe	Mild (S2,S5); Severe (S3,S4,S7); ND(S1)	Severe	Severe (S1) ND (S13)	Mild	Severe	Severe	Severe	Severe (S1)	37	6 Mild/13 Severe
**Behaviour**	+	+	Aggressive, hyperactive, self-mutation	Axiety, anorexy, head stereotypies	ND	ND	Aggressive, progressively improving, hyperactive and chaotic movement	ND	Aggressive, progressively improving	ND	Aggressive, unpredictable mood	ND	ND	ND	ND	7	4 agressive
**Seizures**	Status Epilepticus	ND	EEG abnormalities	ND	-	-	EEG abnormalities	Severe tonic-clonic seizures (S1); Complex partial seizures (S4)	-	Intractable seizures (S1); Epilepsy (S2); seizure disorder (S6); Seizures (S7,S10)	ND	ND	ND	Epileptic seizures	Fever Seizure (S5)	34	12 seizures
**Speech**	Absence	ND	Absence	ND	Delay (Hearing Impairment)	Absence	Absence	Poor development (S3); absence (S7); delayed (S8)	Absence	Delayed (S3,S4,S5,S10)	Normal	ND	ND	Absence	Absence (S1)	35	8 Absence/7 delayed
**Growth or developmental Delay at evaluation**	+	+	-	ND	+	+	+	+ (7/8)	-	+ (S1,S3-9,S10,S14); ND(S13)	-	+	+	+	+ (S1, S2)	37	27 affected
**Spasticity and Contracture**	+	Absence of movement in the 2nd and 5th metacarpo-phalangeal junction	Superior Limbs	ND	-	ND	ND	ND	ND	Joint contractures (S12); contracted thumbs (S10)	Muscle Hypertrophy	Contractures in both little fingers	ND	ND	ND	20	7 affected
**Head**	Microcephaly	Microcephaly	Macrocephaly	ND	ND	Microcephaly	Scaphocephaly	Microcephaly: 4/5 (S1,S3,S5,S7)	Macrocephaly	Microcephaly: 6/9 (S1,S5,S6,S8,S10,S11); Plagiocephaly: 1/9 (S4)	Brachycephaly	Microcephaly	Microcephaly	ND	Microcephaly (S2,S4), trigonocephaly (S2)	36	17 microcephaly/2 macrocephaly/4 other
**Face**	Asymetric	Narrow bifrontal diameter	Long, hypoplasia of the middle third of the face	ND	ND	Asymetric	Long and asymetric. hypoplasia of the middle third of the face	Dysmorphism: 8/8	Long	Short midface (S3)	hypoplasia of the middle third of the face	Small palpebral fissures and wide brow spacing	ND	ND	Dysmorphism, asymetric (S1,S2,S3)	35	20 dysmorphic
**Hair**	Fragile and scarce	ND	Thick and coarse	ND	ND	Fragile and scarce	Thick and coarse. Widow’s peak hairline	Sparse and coarse (S4)	Thick	Sparse hair (S9)	Thick	ND	ND	ND	thin and scarce (S1)	33	9 thick or fragile
**Forhead**	High	Short	High	ND	ND	High	High receding	High or prominent (S1,S4); narrow (S3)	Sloping	Prominent (S5)	Narrow	ND	ND	ND	Prominent (S1,S2)	34	13 affected
**Ears**	dysplasic and low set	ND	Dysmorphic right ear	Dysplasic	Low set	Left preauricular fistula	Low set, prominent antihelix, dysmorphic retrorotated	Low-set ears with thickened helices and lobules (5/8)	Retrorotated	Left ear pits (S1); Low-set (S10)	ND	Auricle thickening ears	ND	Low-set ears	Low set (S1)	36	17 affected
**Eyes**	downslating palpebral fissures	Strabismus	Astigmatism, farsightedness and deep-set	Strabismus	ND	ND	Deep-set	Short palpebral fissures (7/8); hyperopia or astigmatism (S2,S3,S5,S8); coloboma (S4); deep-set (S1)	Hypertelorism, downslanting	Abnormal visual evoked response, cortical visual loss (S1); Small angle esotropia s/p repair, nystagmus (S4); Coloboma (S6); Significant blepharophimosis, difficulty abducting both eyes (S8); Nystagmus, photophobia, exotropia, poor visual motor coordination (S10)	dacryocystitis	Esotropia eyes, an abnormal fit of the lower eyelid and conjunctiva, and blocked lacrimal ducts	Coloboma	ND	Palpebral ptosis, downslanting palpebral fissures (S1,S2); nystagmus and strabismus (S5)	36	24 affected
**Eyebrows**	Thick	-	Thick and synophrys	ND	ND	-	Thick, synophrys	Broad eyebrows with lateral flare (7/8)	Thin	ND	ND	ND	ND	ND	ND	14	11 affected
**Nose**	Bifid nasal tip	-	Bifid nasal tip	ND	saddle nose	Pointed nose	Long and inverted nostrils	Pointed nose with deviation of the tip (S1); prominent nasal bridge (S2); pointed tip of the nose (S5)	High, thin, nasal root	short nose (S3); thin alar base (S5)	ND	ND	ND	ND	bulbuos nasal tip (S1,S2); High, thin nasal root (S3)	34	14 affected
**Palate**	High	Narrow and high arched	High	high-arched	ND	cleft and bifid uvula	high and narrow, bifid uvula	High (S2); narrow (S3)	High	Cleft palate (S9)	High and narrow	ND	ND	high and arched	cleft and ogival (S5)	36	13 affected
**Tooth abnormalities**	Small	Prominent front teeth	Broad and crowded	ND	ND	Oligodoncy, small	Small	prominent front teeth (S3)	Broad and crowded	Teeth missing (S7)	ND	ND	ND	ND	Crowded (S4)	33	9 affected
**Micrognatia**	+	-	+	+	ND	+	+	+ 6/8	+	+ (S7)	ND	ND	ND	ND	+ (S2,S5)	34	15 affected
**Neck**	Short	ND	Short	ND	ND	ND	Short	ND	-	ND	ND	ND	ND	ND	Short (S1)	9	4 short neck
**Chest**	Normal	ND	Visible venous patterm and kyphoscoliosis	Scoliosis	ND	ND	Pectus excavatum and scoliosis	Pectus Excavatum (S3,S5)	Scoliosis	ND	ND	ND	ND	ND	Thoracolumbar scoliosis (S3)	18	8 Pectus Excavatum or scoliosis/11 normal
**Hands**	Right unique palmar crease, bilateral camptodactyly, brachydactyly of the third and fourth finger of the right hand and fourth finger in the left hand, hypotonia	Syndactyly of the 2nd and 3rd, and the 3rd and 4th fingers, short middle phalanges and clinodactyly of the 5th digit at the distal interphalangel joint on both hands, and symphalangism of the metacarpal-phalangeal joints of the 2nd and 5th digits bilaterally	Tapering fingers	Clinodactyly of the fifth finger	sharp fingers and clinodactyly of the fifth finger	Bilateral Camptodactyly	Tapering fingers, camptodactyly of the fifth finger, deep palmar folds	Split hands, oligosyndactyly (S1); Synpolydactyly, camptodactyly (S2); Clinodactyly IV, syndactyly III–IV, tapering fingers (S3); Tapering fingers (S7); Brachymesophalangy, clinodactyly, camptodactyly, tapering fingers (S8)	Tapering fingers	Oligodactyly, 2 digits on right hand, 2 or 3 ulnar sides of metacarpals are missing, 5th finger clinodactyly on left hand (S6); Ectrodactyly (S7); Camptodactyly of all digits (S8); Brachydactyly, mild ectrodactyly, contracted thumbs (S10); Bilateral clinodactyly with bridge or deepset single palmar creases (S11); persistent fetal pads (S14)	Bilateral tapering fingers	showed a transverse palmar crease in the right hand; Little finger of the left hand was deformed	finger-like thumb, mallet index finger, camptodactyly, clinodactyly of the fifth finger and hand dimples at the knuckle.	mild camptodactyly andbrachydactyl. Hypoplastic bilateral middle phalanges of shortfingers	Camptodactyly, bilateral clinodactyly of the fifth finger (S1,S4)	39	25 affected
**Genitals abnormalities**	-	-	Cryptorchidism	ND	ND	ND	Macroorchidism	Hypospadias, penoscrotal transposition (S1); Small labia minora (S2); Small phallus(S3)	-	Hypoplasia of labia minora, proximally inserted anus (S9)	ND	Small penis	ND	ND	ND	28	7 affected
**Feet**	Right equinovarus foot actively and passively redactable	Symphalangism of the metatarsal-phalangeal joint of the 2nd, 3rd, and 4th digits on both feet, with fusion of the middle and distal phalanges of the second and fifth digits and hallux valgus bilaterally	Bilateral sandal sign	partial syndactyly of 2nd,3rd and 4th toes	Bilateral 1st finger duplication	Bilateral equinovarus	bilateral sandal sign, metatarsal hypoplasia	Syndactyly II–III (S1,S2,S4,S5,S6,S8); Long halluces, synpolydactyly, brachydactyly (S3); Long halluces (S5,S6); Syndactyly, sandal gap (S7,S8); brachydactyly (S8)	-	Overlapping toes, flat arches, slender long fingers/toes (S4); cutaneous syndactyly between 2nd and 3rd toes bilaterally (S6); Ectrodactyly (S7); syndactyly of toes 2 + 3 and short 4th and 5th toes bilaterally (S8); 4th toes overlapping 3rd/5th toes, partial syndactyly of 2nd/3rd toes (S9); 2–3 syndactyly, widening at distal toes (S11); Syndactyly of right 4th/5th toes, brachydactyly of left 5th toe, abnormal X-rays of left/right feet (S12)	Sandal sign	syndactylism of the 2nd and 3rd toes of the right foot, a soybean size of ecphyma on the bottom of the left foot, and a superfluous finger on the outer side of the left thumb	rocker bottom feet	Short toes and hipoplastic medial phalanxof the 2nd toe	bilateral syndactyly (S1); sandal sign (S2,S3)	39	29 affected
**Skin**	-	ND	Thin, transparent and hyperlaxyty	ND	ND	Thin and atrophy	-	ND	-	ND	Dry	ND	ND	ND	ND	6	3 dry or thin skin
**Muscle Force alteration**	Hypotonia	ND	-	ND	generalized hypotonia	generalized hypotonia	-	ND	-	ND	ND	ND	ND	ND	generalized hypotonia (S1,S3,S5)	11	6 hypotonia
**Inguinal Hernia**	-	ND	+	ND	ND	+	+	+ (S3)	-	ND	ND	ND	ND	ND	ND	13	4 inguinal hernia
**Genes Involved**	WIPF1, CHRNA1, CHN1, HOXD13, AGPS, PDE11A, PRKRA, DFNB59, TTN, FRZB	Regulatory element far upstream of the HOXD cluster	ZNF533, MYO1B, GLS	ZNF385B, ZNF533, CHD7, DRBI	HOXD	HOXD, DLX1, DLX2	NTCR	HOXD (patients with hand anomalies, except 8); DLX1/DLX2 (micrognathia and cleft palate); CHN1 (ocular anomalies)	HOXD, MTX, ZNF804A, CERKL, NEUROD1, FRZB	HOXD (patients with limb anomalies); CHN1 (ocular anomalies)	NTCR, GLS, ZNF804A	GAD1, DCAF17, SLC25A12, ITGA6, HOXD, DLX1/DLX2, CHN1	HOXD, SHFM5, DLX1, DLX2	HOXD, ZNF385B	HOXD	-	-

**Fig. 2:**
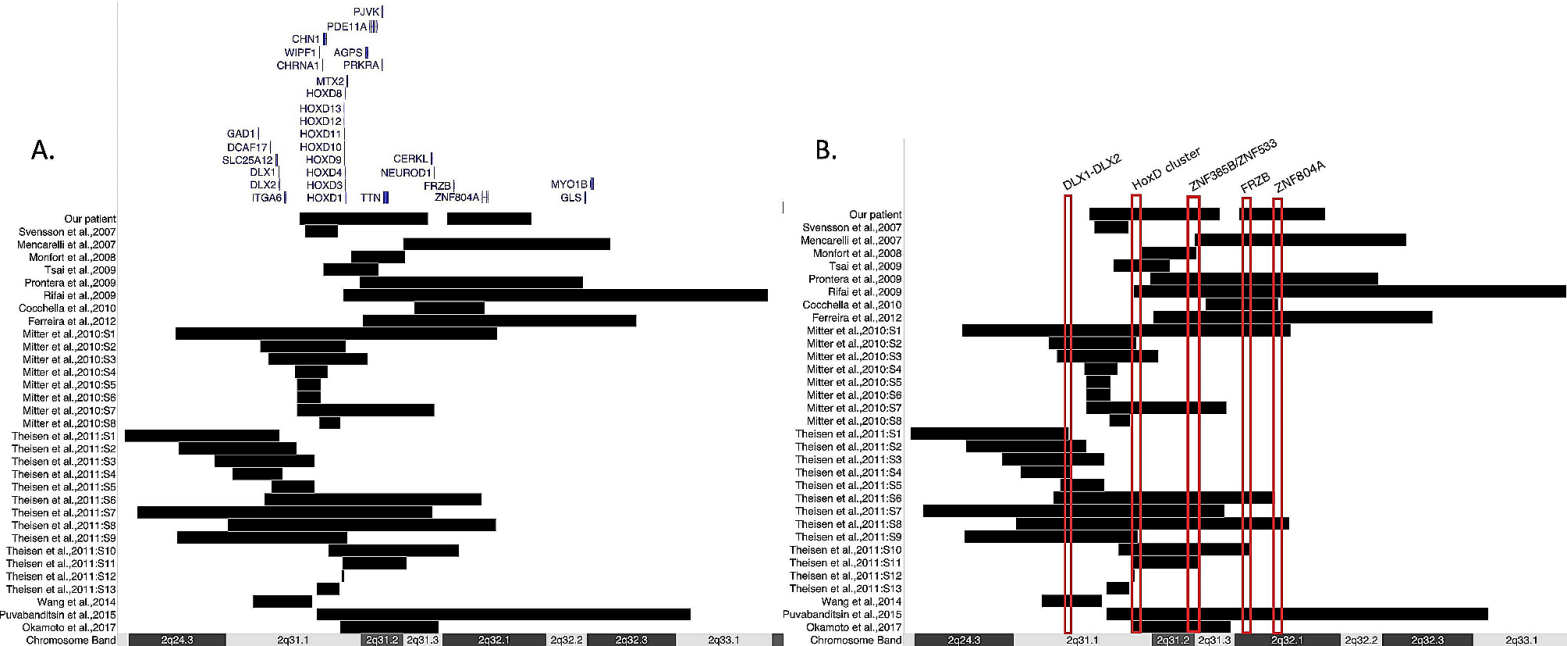
Map of the deletions, comprising at least the 2q31.1 region, of the cases found in the literature. The map was create using UCSC Genome Browser on Human (GRCh37/hg19) [20]. Genomic coordinates of the reported deletions were converted to the GRCh37/hg19 coordinates using the LiftOver tool of the UCSC. Black bar indicates the length of the deletion found. Delimited regions indicate some genes or clusters mentioned in the text. Patients from Dimitriv et al. [3] were not included as no genomic positions were reported. Panel A displays the genes reported in both the literature and our case study, whereas Panel B highlights the most relevant genes according to the literature review, including those occurring with higher frequency

Comparison of our patient’s clinical findings with those in the literature (see references above, Table 1) shows concordance with the characteristics of 2q31.1 microdeletion syndrome. The deletion identified in our patient spans a genomic segment common to multiple cases previously reported (Fig. 2), including genes such as *FRZB* and the *HOXD* cluster. The *HOXD* genes are essential for embryonic development, especially in cell-to-cell communication, and are implicated in the early development of mammalian limbs [7, 15]. In our case, 61 genes have been compromised within the microdeletion (Fig. 1), including a series of *HOXD* genes: *HOXD13*, *HOXD11*, *HXOD10*, *HOXD9*, *HXOD8*, *HOXD4*, *HOXD3*, and *HOXD1*. The absence of the *HOXD* cluster, documented in 15 out of 38 cases from the literature ( or 16 out of 39 when our proband), has been consistently associated with limb anomalies (Table 1; Fig. 1). Thus, the camptodactyly and brachydactyly seen in our patient are likely due to the loss of these homeotic genes, as similar symptoms have been observed in other instances of the syndrome with an absent *HOXD* cluster [2, 4].

The long arm of chromosome 2 has been identified as a potential candidate region associated with autism, primarily due to the presence of critical genes involved in development. Patients with deletions in this region may present with a range of manifestations, including speech impairment and behavioral disturbances [14, 16]. Individuals diagnosed with the 2q31 microdeletion syndrome, such as the case described here, often exhibit behavioral disturbances that may include symptoms of aggression, anxiety, mood instability, hyperactivity, and chaotic movements. Among the genes deleted in our patient, *PRKRA* is of particular interest due to its role in cellular stress response and synaptic plasticity, which are crucial for learning and memory processes [17]. Notably, though disturbances in this gene were observed, the absence of PRKRA alterations in some patients presenting behavioral anomalies precludes drawing definitive conclusions about its involvement. Moreover, additional genes implicated in the behavioral, speech, and neurological manifestations of the 2q31 deletion syndrome include ZNF804A and ZNF385B, which encode zinc-finger proteins linked to mental and neurological abnormalities associated with the syndrome [5, 14] (see Fig. 2).

However, our findings indicate that while numerous patients with severe cognitive impairment or behavioral disturbances, including our patient, have deletions that encompass these genes, there are other with similar manifestations who do not show recognizable genetic alterations in these areas. This variation suggests that additional elements, possibly regulatory sequences adjacent to these genes or at other loci, might influence the neurological phenotype seen in patients with 2q31-2q33 microdeletions. In the case reported here, MRI disclosed dysgenesis of the corpus callosum, encephalomalacia in the right frontal lobe, retraction of the frontal pole of the lateral ventricle, and periventricular hyperintensities, which further elucidate the structural brain anomalies linked with the 2q31.1 microdeletion syndrome.

Furthermore, the presence of the TTN gene within the deleted region is intriguing, as it is not currently linked with any clinical outcomes in this syndrome. However, autosomal dominant cardiomyopathy due to haploinsufficiency of TTN has been documented [18]. Consequently, the contribution of the TTN gene to the clinical phenotype in our patient remains unclear. Of 11 cases with deletions involving the TTN gene, seven have incomplete information regarding heart defects-it is unknown whether these patients were asymptomatic or if cardiac evaluation were not conducted. Two of the patients showed no cardiac abnormalities, while the remaining two presented with mild hypertension, bicuspid valves, and atrial and ventricular septal defects. Notably, heart defects were observed in five patients without TTN gene alterations. These findings imply that other genes may also contribute to the cardiovascular manifestations observed in these patients.

In cases where patients exhibit the velocardiofacial phenotype without confirmed diagnosis of 22q11 microdeletion syndrome-one of the most prevalent chromosomal deletions associated with congenital heart diseases in humans [19]-a presumptive diagnosis of 22q11 microdeletion syndrome is typically considered. For our patient, f FISH analysis was performed to detect alterations in the 22q11 cytoband, which yielded negative results. Consequently, high-resolution aCGH analysis was undertaken, revealing a discontinuous deletion in the 2q31-2q32 regions. This findings highlights the importance of employing techniques that offer high resolution and comprehensive DNA coverage for the diagnosis of complex syndromes.

Furthermore, the use of CGH has facilitated the improved characterization and precise delineation of deletion breakpoints in DNA. This has led to the identification of distinct syndromes associated with these regions, including the 2q31.1 microdeletion syndrome, 2q31.2q32.3 microdeletion syndrome, and 2q33.1 microdeletion syndrome [10]. However, in our patient´s case, the deletion encompasses all 2q31 cytobands and a substantial portion of the 2q32.1 cytoband. This renders her phenotype, and the specific genes involved, more complex compared to other reported cases.

## Conclusions

In conclusion, our comprehensive case report details a patient with a complex, discontinuous heterozygous deletion spanning the 2q31.1 to 2q32.1 cytobands. To our knowledge, this is the inaugural documented case in the literature of a discontinuous deletion in these particular genomic regions, presenting significant challenges for diagnosis, management, and prognosis. Our systematic review of the literature on cases with deletions involving at least the 2q31.1 cytoband yields valuable insights into genotype-phenotype correlations, shedding light on the frequency and clinical manifestations observed in these patients.

Furthermore, our study underscores the utility of high-resolution CGH techniques in diagnosing complex and rare disorders. The CGH method enables the detection of an expanding spectrum of genomic alterations, while also facilitating the precise identification of affected genes with exceptional resolution. This contributes to a better understanding of the underlying genetic mechanisms involved in the pathogenesis of these conditions. Moving forward, continued research and collaboration are needed to further characterize the genotype-phenotype relationships and delineate the full spectrum of clinical symptoms associated with deletions involving the 2q31.1 cytoband and adjacent regions. This knowledge will aid in enhancing diagnostic accuracy, prognostic assessments, and therapeutic interventions for affected individuals, ultimately improving patient outcomes in the realm of rare genetic disorders.

## Data Availability

The datasets used or analyzed during the current study are available from the corresponding author on reasonable request.

## Abbreviations

CGH: Comparative genomic hybridisation
CPAP: Continuos positive airway pressure
MRI: Magnetic Resonance Imaging
FISH: Fluorescence in situ hybridization
WIPF1: WAS/WASL Interacting protein family member 1
CHRNA1: Cholinergic receptor nicotinic alpha 1 subunit
CHN1: Chimerin 1
AGPS: Alkyglycerone Phosphate Synthase
DFNB59: Pejvakn
HOXD: Homebox D
PDE1A: Phosphodiesterase 1 A calmodulin-dependent
cAMP: Cyclic adenosine monophosphate
cGMP: Cyclic guanosine monophosphate
PRKA: Phosphoribulokinase
TTN: Titin
IQR: Interquartile Range
